# “It’s a Bit of a Double-Edged Sword”: Motivation and Personal Impact of Bereaved Mothers’ Advocacy for Drug Policy Reform

**DOI:** 10.1177/10497323211006383

**Published:** 2021-09-16

**Authors:** Heather Morris, Elaine Hyshka, Petra Schulz, Emily Jenkins, Rebecca J. Haines-Saah

**Affiliations:** 1 University of Alberta, Edmonton, Alberta, Canada; 2 Moms Stop the Harm, Victoria, British Columbia, Canada; 3 The University of British Columbia, Vancouver, British Columbia, Canada; 4 University of Calgary, Calgary, Alberta, Canada

**Keywords:** drug policy, advocacy, family, substance use, harm reduction, bereavement, qualitative narrative, Canada

## Abstract

North America’s overdose crisis is an urgent public health issue that has resulted in thousands of deaths. As the crisis began to take hold across Canada in 2016, bereaved parents, mainly mothers, emerged as vocal advocates for drug policy reform and harm reduction, using their stories to challenge the stigma of drug-related death. In 2017, we launched a qualitative research partnership with leading family organizations in Canada, conducting interviews with 43 mothers whose children had died from substance use, to understand their experiences of drug policy advocacy. Our findings showed that participants’ motivations for engaging in advocacy were rooted in their experiences of grief, and that advocacy led to feelings of empowerment and connection to others. Our research suggests that advocacy can be cathartic and associated with healing from grief, but that “going public” in sharing a family story of substance use death can also have a considerable personal cost.

## Introduction

The overdose crisis has been described as one of the most urgent and challenging public health issues of our time (Alpert et al., 2019; Tyndall, 2018). In Canada, more than 17,000 people died from an apparent opioid toxicity death between January 2016 and June 2020, with provinces in Western Canada continuing to be most directly impacted (Special Advisory Committee on the Epidemic of Opioid Overdoses, 2020). The United States has also continued to experience a crisis, with 81,230 deaths attributable to drug overdoses between June 2019 and May 2020 and an increase in mortality now associated with the COVID-19 pandemic (Centers for Disease Control and Prevention, 2020). The overdose epidemic has complex causation, including aggressive marketing practices of pharmaceutical companies (Alpert et al., 2019), opioid overprescribing and diversion (Dhalla et al., 2009; Gomes et al., 2011), the introduction of highly toxic novel synthetic opioids into drug markets (Hedegaard et al., 2020), and proximal and distal social determinants that place subpopulations of people at greater risk of overdose death (Dasgupta et al., 2018). Prominent professional organizations in Canada representing health care providers, policymakers, and researchers as well as nongovernmental organizations (NGOs) have endorsed a public health approach to overdose that includes the meaningful involvement of those with lived and living experience in program planning, policymaking, and delivery of interventions (Canadian AIDS Society, 2015; Canadian HIV/AIDS Legal Network, 2005; Dickson-Gomez, 2010; Friedman et al., 2012; Golovanevskaya et al., 2012; Jozaghi, 2014; Jozaghi et al., 2018; O’Gorman et al., 2014).

An emerging perspective that has been increasingly visible since 2016 in North American drug policy discourse, but which has not been studied empirically, has been that of bereaved parents who have lost a child to substance use. Three volunteer advocacy organizations representing families who have been impacted by substance use in Canada are Moms Stop the Harm (MSTH; est. 2016), Moms United and Mandated to Saving the Lives of Drug Users (mumsDU; est. 2015), and The Voice of the Family (est. 2016; the latter group being an advocacy organization whose leadership has since retired from advocacy). MSTH and mumsDU use various methods to advocate for drug policy reform, including public speaking, organizing public protests, media engagement, meetings with policymakers and politicians, and working collaboratively with researchers to advance evidence-based drug policy reform. Both grassroots volunteer organizations were initially created to advocate for family members who have lost a loved one to substance use, but have since expanded to provide peer support for individuals whose loved ones have died or are currently experiencing substance-related problems. The political advocacy of both groups is unique due to its outspoken support for harm reduction philosophy and interventions, an approach that seeks to minimize the harms associated with substance use while supporting individuals who may not want to abstain from drug use (Kerr & Ti, 2013).

While bereaved parents have emerged as significant actors in Canadian drug policy reform, a key element missing from discussions to date has been what motivates parents to undertake volunteer advocacy work and the personal toll it has for them. Studies of parents whose children live with disabilities suggest that motivational factors for parental advocacy may involve a sense of personal obligation (Wang et al., 2004), altruism (Woodgate et al., 2008) and a disappointment in and subsequent desire to improve services for other families and individuals (Ryan & Runswick Cole, 2009; Wang et al., 2004; Woodgate et al., 2008). Parents from this research have been positively impacted by their advocacy activities through gaining a sense of self-satisfaction (Milliken, 2001; Ryan & Runswick Cole, 2009); enhanced coping, a sense of control, or empowerment (Boshoff et al., 2016; Ryan & Runswick Cole, 2009; Wang et al., 2004); and gaining a feeling [understanding] of altruism (Phelps et al., 2009). However, parental advocacy may also contribute to fatigue (Duquette et al., 2012; Milliken, 2001), stress (Duquette et al., 2012; Wang et al., 2004), financial and career pressures (Duquette et al., 2012; McCabe, 2007), and guilt (Duquette et al., 2012). Important to note here is that much of the work on parental advocacy in the disability field has focused on parents advocating for the needs of their own child, as opposed to policy advocacy at a population level.

When thinking of parent advocacy about substance use, Mothers Against Drunk Driving (MADD; El-Guebaly, 2005; Sweedler, 2006) is a likely referent. What was once a grassroots movement has grown to be a highly visible, North American advocacy organization that advocates for family/victim support, increased penalties for impaired driving, legislative changes associated with minimum drinking ages, sobriety check points, liability laws for servers of alcohol, and changes to social norms regarding impaired driving (El-Guebaly, 2005; Sweedler, 2006). Empirical evidence has documented that the advocacy by MADD is correlated with reducing alcohol-related driving fatalities (Asbridge et al., 2004; Fell & Voas, 2006); however, research exploring the motivations guiding the work of MADD members, as well as the personal implications of such activities for its members, is lacking. In addition, although the efforts of MADD appear to bear a close resemblance to those of MSTH and mumsDU, two substantial distinctions exist. First, the parents represented by MADD have most often experienced the death of a child due to the conduct of another person. In contrast, owing to stigmatizing (mis)perceptions about addiction, including that it is a “personal choice,” the children of mumsDU/MSTH members are perceived by some in society as partially culpable for their own death. Second, MADD’s messaging has focused on interventions aimed at stigmatizing impaired driving in an effort to minimize the behavior and supporting victims of drunk driving, whereas mumsDU and MSTH are advocating for prevention, treatment, and harm reduction approaches to supporting people who use drugs.

The grief literature has provided few studies that report on the emotional impact to parents of being engaged in advocacy activities following the death of a child from substance-related causes. Family members have indicated that advocacy contributes to personal growth (Feigelman et al., 2020), meaning-making (Titlestad et al., 2019), and an enhanced sense of purpose (Nowak, 2015). However, such studies have focused more on the grief experience than on public health advocacy and rarely explored the motivations behind these efforts. As a result of these gaps in the scientific literature, our research was aimed at addressing the following question:

**Research question 1 (RQ1):** What are the motivations behind and the personal impacts experienced by bereaved mothers who advocate for drug policy reform?

## Method

This article draws on data from a larger qualitative project, the purpose of which was to systematically collect, analyze, and share the stories of Canadian mothers who have experienced the death of a child to substance use–related causes and who have subsequently engaged in advocacy on substance use issues. For the purposes of this project, advocacy was defined broadly to include a wide range of formal and informal activities to change attitudes or promote policy change. We defined substance-related death as a death related to a person’s use of substances (e.g., due to overdose or drug poisoning, suicide, physical health complications, or unmet health care needs due to substance use). Participants were required to be 18 years of age, live in Canada, and have first experienced a child’s death a minimum of 6 months before the interview.

Our project protocol received approval from the research ethics boards at the University of Calgary, University of Alberta, and the University of British Columbia. Between June and November 2017, we conducted a total of 43 in-depth, semi-structured interviews with women from British Columbia (*n* = 17), Alberta (*n* = 12), Saskatchewan/Manitoba (*n* = 4), and Ontario and the Maritime provinces (*n* = 10).^1^ Narrative interviewing techniques were used to support close examination of both the structure and content of mothers’ stories related to advocacy (Wells, 2011) while accounting for the influence of social and cultural context (Patton, 2015). Efforts were made to maximize regional and sociodemographic diversity, family context, and level of advocacy involvement as much as possible, and we chose to solely recruit mothers, given their prominence in the Canadian public discourse at the time. Most participants had a household income of greater than Can$50,000 (70%) and reported their ethnicity as White (95%). A total of 45 children had passed away as two of the participants we interviewed lost more than one child to substance-related death. Further characteristics of our sample are presented in Table 1 in Supplemental File A. Our community partners representing mumsDU, MSTH, and the Voice of the Family contributed to designing the study, developing the interview guide (Supplemental File B), background questionnaire, and participant recruitment through distribution of an information letter through their social media and email networks. Upon receiving a letter of invitation and reaching out to the study principal investigator (PI) (R.H.-S.), participants were contacted by the PI or one of two regional research assistants to arrange for an individual meeting in person or by telephone. All interviews were audio-recorded, transcribed verbatim, and lasted between 26 and 135 min. Field notes were generated by the study team following each interview to record context and key insights. The majority of interviews (84%) took place in person, with the remainder being conducted over the phone. Participants provided their informed, written consent prior to the start of the interview and received a Can$50 gift card to recognize their time and contributions once the interview was complete.

We employed multiple strategies to ensure both methodological and analytic rigor, including prolonged engagement with study participants, ensuring thick description from participants, member checking of interview transcripts, negative case analysis, co-coding a subset of transcripts, use of a detailed audit trail to document group decision-making/analytical decisions, and using a reflexive approach during analysis (Cohen & Crabtree, 2006; Forero et al., 2018; Lincoln & Guba, 1985; Morse, 2015; Richards & Morse, 2013). Saturation of data was determined to have been reached once it became evident that participants were presenting very few new ideas and when negative cases had been sufficiently explored (Mayan, 2009; Richards & Morse, 2013). NVivo 12 was used to organize the data, and Braun and Clarke’s (2006) thematic analysis procedure guided qualitative data analysis. Specifically, H.M. read all the transcripts and generated an initial list of broad initial codes (both inductively and deductively from the interview guide) in collaboration with R.H.-S., who co-coded 10% of the interviews. Coding discrepancies were resolved by consensus (Morse, 1997; Patton, 2015). A total of 10 block codes were developed, including Advocacy Structure, Personal Impact, Family Impact, Community Impact, Substance Use Treatment and Relapse, Broken Systems, Media, Motherhood/Gender, Story/Narrative, and Other).

We describe data contained in the Advocacy Structure (Personal Impact and Motivators) code for the purposes of this article. As per Braun and Clarke (2006), transcripts were read through multiple times (while taking memos), with codes generated by grouping relevant phrases, words, and concepts together. From here, we developed categories and subcategories, which were refined over time. Broader themes were subsequently developed with a “thematic map” generated to ensure that coded material fit both “within” and “across” the overall themes that were developed. In this article, we explore two key themes generated through our analysis: (a) from grief to honor: the motivators of bereaved mothers’ drug policy advocacy, and (b) the double-edged sword of advocacy.

## Results

### From Grief to Honor: The Motivators of Bereaved Mothers’ Drug Policy Advocacy

Bereaved mothers’ motivations for engaging in advocacy were closely tied to the emotions that arose from their experiences related to grief. While stories included tremendous disappointment and despair, many participants spoke of their passion for advocacy being born out of the pain of losing a child. As one participant expressed,

And I think maybe, too, in the beginning, there was a little bit of, “If I do good things, maybe she’ll come back.” You know? Like that sort of irrational grief sort of stuff that comes into it. . . .

In addition to grief, nearly half of participants also spoke of a tremendous sense of anger that motivated their advocacy—anger that their child died prematurely, anger at how the health care system or justice system had failed their child, and anger at the stigma that exists toward people who use drugs and their families. Some expressed anger toward themselves because they were not able to “save their child,” which could lead to a lack of self-confidence in their advocacy activities. These feelings of anger would often prompt mothers to write a letter to a politician, reach out to other key stakeholders, or speak to the media. One participant recounted her anger upon hearing that there had been multiple deaths in a First Nations community over a 2-week period and that “nobody was telling their story,” while another participant expressed,

Like, so many people said, “Oh, you’re so courageous.” And I thought, “That’s not courage. I was pissed.” I was angry. I’m angry. And I think anger is not a bad thing. It’s where we get change.

Other participants reflected on being motivated to engage in advocacy after spending time with others with shared experience or through support from family or community members. One such example came from a participant, who commented that “. . . it was really my daughter who pushed us into this.” Motivation from the community also came from seeing positive changes or outcomes resulting from one’s advocacy activities. One participant, for example, recounted that members of her advocacy organization were encouraged after having gained access to meetings with high-level officials, including the Canadian Prime Minister, the Federal Minister of Health, and the Canadian Ambassador to the United Nations. For others, the motivation to advocate fulfilled a personal need or responsibility to oneself or helped them cope with grief. A participant commented that following her son’s death, she had expressed to herself, “. . . at first, it was sort of like, okay, [Son]’s gone. Now what? Now I need to do something. Right? And it was my way of avoiding. I think it was a total avoidance at first.” In addition, a few mothers spoke of being motivated by working to erase the shame and stigma:

And that’s part of why I tell the story I think, deep, deep down, is I want to erase that shame for myself by telling my story and that shame for other people by telling my story. “Well, if she [another bereaved mother/advocate] can say it, maybe I can say it . . . .”

Participants regularly reflected on being motivated by a sense of hope. Although some spoke in more general terms of hoping for change or making the world a better place, others spoke more specifically of their hope for health care system reform or that the silence and stigma around substance use disorder would eventually be broken. Many also expressed that they were motivated by hope for fewer deaths or that other parents would not have to experience the same loss that they had gone through. Such was the case with one woman, who stated,

. . . I think I have a responsibility to try and change things so that this will not happen again to someone else. Because it’s not right, it’s very, very wrong, and I don’t want to be an accomplice to a tragedy.

Quite a few of the women expressed this sense of social justice or responsibility to others as a motivating factor, many of whom spoke of wanting to help people and “do the right thing.” In one such example, a participant reached out to the media when her daughter was actively using drugs (before her subsequent death) after hearing about the toxic drug supply on the streets. A sense of concern and responsibility drove her to alert the public through the media to prevent future deaths. A different participant also alluded to this sense of responsibility when she stated, “. . . I’d rather not be doing this. I’d rather be doing anything else. But your sense of duty and your responsibility to serve others and yourself in some manner, kind of trumps that reluctance.” Another participant took this sentiment one step further in saying, “How would I ever look at another mom down the road who just lost their child through something like this if I didn’t do something?”

Finally, one of the most significant motivators for advocating was taking the opportunity to honor one’s child. Many spoke of how advocacy gave them a “reason” for talking about their son or daughter, which can sometimes come to a premature end with friends or family members after the initial period of grieving. Being given the opportunity to say their child’s name through advocacy meant that their child was not remembered for how they died, but rather for how they lived. While one participant who lost two children to substance use shared, “. . . everything I do, I do because of them,” a different participant spoke of how her motivation to advocate came partly from wanting to preserve her child’s memory, stating, “I’m so afraid of not remembering events or not remembering [Son], or forgetting what he looked like. . .” On the contrary, one woman spoke of a conversation she had with her daughter about the severity of her cravings for the substances that later contributed to her death. She reflected on this when speaking of what motivates her to advocate:

And I always say I’m not doing it for [Daughter]. I’m doing it for somebody else’s child. That’s actually a lie. It’s a blatant lie. I am doing it for [Daughter]. I’m doing it because she was desperate. And as a mother, you never want to see your child desperate for anything. So I couldn’t give her the drugs in those situations. But I can give her resolve.

### The Double-Edged Sword of Advocacy

The personal impacts of advocacy were both constructive and helpful, but also, at times, harmful or damaging. Specifically, for participants in our study, the personal impacts of their advocacy centered on three experiences: Enhanced Sense of Empowerment, Solace in Connecting with Others, and the Juxtaposition of Advocacy (Pain vs. Healing)

#### Enhanced empowerment

Advocacy brought with it an enhanced feeling of empowerment for nearly every participant. Over half of the women discussed a sense of personal learning or growth that accompanied their advocacy activities. For example, participants learned about substance use and mental health (and resources for both), treatment, stigma, harm reduction, grief, and advocacy itself. In some instances, personal opinions began to change about certain concepts or practices with advocacy activities. One participant stated, “before my son’s death. . . I thought, like so many others, it was a choice, and I was so wrong,” whereas a different participant expressed,

. . . if you want to talk about radicalization in a positive way, being part of our group radicalizes a lot of people. People come in thinking that every dealer should be arrested, and then they feel the most important thing is that we decriminalize. . . .

In addition to changing attitudes about particular issues, many participants started to learn more about themselves over time. One woman exemplified this when she stated, “. . . I’m learning a lot about myself, what I’m capable of and where I’m strong and where I need to change and what I need to improve. So it’s meaningful in that way.”

Many participants expressed that advocacy brought with it enhanced feelings of strength, resilience, or confidence. This was significant as it was not unusual for mothers to feel a sense of powerlessness when their child was alive and active in their addiction. One participant stated that advocacy had allowed her to take an opportunity that “. . . has brought me to my most powerful self, to my most true self.” Similarly, another woman expressed that advocacy contributed to making her both brave and strong while reflecting on a conversation she had with a well-known politician and member of the cabinet who told her, “. . . don’t ever stop telling your story because telling your story will create change.” Feelings of resilience often translated to a sense of pride and respect for oneself as it did for a participant who stated, “. . . this almost feels like I’ve found my young, radical side again, and I like that side of me.” However, two of the participants spoke of feelings of guilt that accompanied appreciating the positive attention that came with advocacy activities, with one saying that her therapist helped her resolve such emotions.

Advocacy provided participants with a sense of meaning or purpose and a feeling that one is making a difference. One woman expressed that it was because of this sense of purpose “. . . that I feel more alive than I did before when I was just functioning,” whereas another stated, “. . . this is my defining moment. I don’t want it to be anyone in my family’s defining moment, but it is mine.” Finally, participants became empowered through the comfort or sense of freedom that came with being open and honest about their child’s substance use, which was not always possible to do when their child was alive. Participants also felt empowered through a sense of hope generated through their advocacy activities—hope that their efforts will bring about changes that will ultimately improve others’ lives and hope that advocacy will help with their own grief process and personal journey. One participant exemplified such hope when she spoke about realizing that her son was no longer physically present with her. “So that little piece of hope, that’s gone. . . you have to hang on to some hope for something else. So I think that’s part of what advocacy is . . . without that you can’t keep going.”

#### Solace in connecting with others

Nearly all the participants expressed that advocacy brought with it a tremendous opportunity to connect with others, either with other bereaved mothers or with members of the community. As indicated above, many of the participants spoke of the strong relationships they had developed within their respective advocacy organizations due to a shared sense of loss. One woman stated that, at the beginning,

. . . I didn’t know for sure if going down that path with those women was going to resonate with me ultimately. But every step forward with those women proved to me that they absolutely knew what they were talking about. . .

Another participant, like many of the participants we interviewed, spoke highly of the advocacy leaders, stating, “. . . they have basically kept me going.” Others connected with bereaved mothers who were not necessarily involved in advocacy activities but who they had met through community events or bereavement work. However, fostering a sense of connection could pose a challenge for those who lived more rurally, although online platforms helped with this.

Participants also expressed that advocacy brought with it a connection between themselves and other members of the community, including friends, family, strangers, their child’s peers, and health care providers. One woman spoke of sitting with people who used drugs in her community to ensure their safety and being called to administer naloxone to someone who was overdosing and reluctant to call the police. Another participant, on the contrary, recounted the time that a harm reduction manager tried explaining to her the vital contribution she was making to the substance use policy context by stating, “You know, I can speak, and people hear. . . You speak, and people feel.” But such connections at times extended further afield to include police, news reporters, researchers, and elected officials. One woman spoke of a provincial Minister of Health being brought to tears during a meeting with bereaved mothers while another witnessed a high-ranking government official tearing up during an in-person meeting she attended. Seeing others express emotion through tears brought with it a sense of comfort to bereaved mothers and a hope that their advocacy was impacting others in a meaningful way.

Finally, nearly half of the participants spoke of advocacy, enhancing a feeling of closeness with their child who had passed away. Several mothers indicated that their children would have been proud of the work that they were doing, or that they had felt their child’s presence during their advocacy activities. One woman confided, “And honestly, crazy as it sounds, I feel him pushing me,” while another expressed, “. . . that the energy that was [Son] is now my energy. . .” A different participant agreed that her advocacy helped to maintain a connection with her child and that this was a primary driver of her work:

And I think that advocacy work maintains your commitment to that relationship, and I think it’s important . . . You’re publicly saying, “I’ve lost my physical child, but I have not lost my commitment to my child.” And I think that’s important for mothers to say.

While this sense of connection with their child was prominent for some, others were unsure or did not feel that same relationship between advocacy and the closeness they felt with their child. Some indicated that their children were very private and may not have been happy with their mother’s decision to advocate and share their story, whereas others felt that they connected with their child in other ways outside advocacy. One participant expressed that she has always felt deeply connected to her child but that her advocacy helped keep him connected with other people in her community and extended family members.

#### The juxtaposition within advocacy: Pain versus healing

Participants expressed that advocacy brought with it both pain and healing, which ultimately created a profound juxtaposition for some. While several participants were motivated to engage in advocacy to find healing, the perceived impact on their well-being was mixed. On one hand, advocacy work was emotionally difficult or draining for nearly all participants. As such, it had the potential to impact well-being and exacerbate feelings of grief and loss negatively. Mutual support (in person or online) was an essential component of many mothers’ advocacy activities; however, it also had the potential to leave participants feeling extremely upset and emotionally drained afterward. One participant stated that advocacy kept her in a “. . . perpetual place of grief. If it’s not for your own situation, it’s for someone else’s.” Others spoke of advocacy contributing to strained relationships with family members, having to endure hurtful and stigmatizing comments from the public at times, and a “wave of grief” that can follow media engagement or large advocacy events:

Sometimes I wonder how long I can do that [advocacy]. . . you sort of open up wounds a lot of the time with media work, with sharing stories, and with speaking to newly bereaved parents. That probably opens up the most wounds. They tell their story . . . and it brings back sort of this “waking up in the middle of the night in a panic” [feeling]. . .

Many participants spoke of the need to occasionally “step back” from advocacy activities to protect oneself emotionally to counteract these and other advocacy stresses. Tensions could also arise with other advocates, contributing to stress and strained relationships within the advocacy organization:

. . . we all have opinions on how things should be done. . . there’s those that want to kill with niceness and get it done that way. There’s those that want to just yell and scream and hold their signs, and there’s those that believe in things that others don’t believe in . . . and sometimes that makes it quite difficult.

However, a contrast arose as the pain of advocacy intersected with its potential to contribute to healing. One participant stated,

I think that it’s kind of flip sides of the coin. . . on one hand, the advocacy can be cathartic and healing. . . and then I think on the other side. . . it’s the right thing to do, but it just hurts.

A different participant, alternatively, likened the experience to “. . . always pulling a Band-Aid off. But I would think after a period of time that your skin gets toughened up to that a little bit.” Another participant added to these sentiments, stating, “And sometimes, some of the questions I’m asked really expose the pain but in a good way. . . It hurts but it feels good.”

Thus, although there is an emotional cost to advocacy work, nearly all the participants spoke of advocacy as helping to heal, often acknowledging that grief would always be present. The mutual support that occurs between members of an advocacy group was described as one of its most important benefits. Participants reflected on how such groups allow people to think about, discuss, and process their child’s substance use and subsequent death with others who have undergone a similar experience. Although some of the members (e.g., those living more remotely) may have only interacted online with others, the support they received from fellow advocates was deemed significant. While one participant spoke of “regaining strength by being with others,” another stated, “. . . I’ve told these women things that I haven’t even said to my husband . . . because they get it.” In a similar vein, advocacy brought healing in the form of helping others, including friends, family members, their child’s friends, or members of the community. To be able to do this while channeling something negative into something positive, speaking their child’s name and keeping their memory alive brought comfort to many, including one woman who stated, “. . . I think we all advocate because we want to honour our children. But at the same time, we also want to be able to help somebody else.”

## Discussion

The findings of this study highlight that mothers’ motivations for advocacy were multifaceted and strongly rooted in their experience of grief. The motivation for this advocacy work often came from a deeply personal place where, in many cases, participants experienced anger, intense grief, and immense disappointment in how their child was treated within a system that was expected to care for them and keep them alive. As such, the participants we spoke to worked tirelessly in their messaging to address the structural and social determinants of substance use and the important role of the state in supporting the lives of all people who use drugs through anti-stigma efforts, enhancing harm-reduction efforts and the creation of new laws and policies that support decriminalization and the legal regulation of substances. Bereavement associated with the experience of a family member’s death due to substance use is unique and associated with feelings of anger, relief, guilt, shame, and stigma (da Silva, 2007; Feigelman et al., 2011; Templeton et al., 2016; Titlestad et al., 2019, 2020; Valentine et al., 2016; Walter et al., 2017). It comes as no surprise then that while the participants in our study expressed that advocacy can promote healing and enhance personal empowerment and connectedness with others, this often came at an emotional price. Our findings echo those from the extant grief literature that describes creative actions and advocacy activities undertaken by family members, which, in some cases, helped to contribute to the bereaved making sense of their loss (Titlestad et al., 2019) and posttraumatic growth (Feigelman et al., 2020). Nowak (2015) conducted a grounded theory study that explored the grief experience of eight parents in the United States, whose child passed away from a drug-related death, with one of the themes pertaining to “transforming identity.” This theme included activities that would protect others from experiencing a loss similar to what they had endured, minimize the social stigma of people who use drugs, promote harm reduction, educate the public about substance use, and advance initiatives that would minimize opiate use by others and prevent further deaths. Ultimately, parents were transformed following their child’s death

. . . by a process that brought meaning to the death in a way that honored . . . [their child] . . . and through the discovery of a purpose that ensured a continued and heartfelt relationship with . . . [their child]. . . (Nowak, 2015, p. 112)

Our research aligns with the notion of relational empowerment put forth by those who have described it as a process of acquiring power that is both collective and transformative, “. . . intended to alter structural conditions and dynamics in social, political and community contexts” (Christens, 2012). Woodall et al. (2012) have argued that the term “empowerment,” which emphasizes both process and outcomes, has, in recent years, been diluted and misrepresented from its original roots within the social justice literature. This is partly due to an emphasis on the individual within a neoliberal political and policy environment, particularly in the area of public health and health promotion. As such, “this clearly offers challenges to promulgating the original tenets of achieving empowerment which advocates shared experiences of powerlessness and community mobilization and organization” (Woodall et al., 2012, p. 743). In this regard, Christens (2013) has argued that transformative power and change develop through cognitive, behavioral, and emotional processes and relationships. Critical elements of this relational component of psychological empowerment include collaborative competence, bridging social divisions, facilitating others’ empowerment, mobilizing networks, and passing on legacy (Christens, 2013). While it is beyond this article’s scope to discuss each of these elements related to bereaved mothers’ advocacy, it is worth acknowledging that all are essential to the continued efforts put forth by members of MSTH and mumsDU. Thus, while the participants we spoke to clearly articulated feelings of personal growth, enhanced knowledge, and increased strength and resilience as a result of their advocacy, this increase in personal capacity did not occur in a social vacuum but rather as part of a larger process, generated by their exchanges with significant others both within and outside of their grassroots organizations.

In undertaking this study, our intent was not to overshadow or undermine the long history of advocacy that has been launched by people who use drugs and other people with lived experience but instead add to the voices of those who have been most directly impacted by substance use. Similar to the participants in our study, members of the Vancouver Area Network of Drug Users (VANDU) have expressed that advocacy has brought with it a sense of empowerment (Jozaghi, 2014) and purpose, as well as an opportunity for “drug users to view themselves in a more positive light that stands in stark contrast to the disabling stigma imposed on drug users by society” (Kerr et al., 2006, p. 67). Kerr et al. (2006) have also acknowledged that advocacy work with VANDU has been credited with helping members be more conscientious about the environment and their health. More recently, participants in a study by Bardwell et al. (2018) spoke of an increase in status and community acknowledgment, increasing structure and control in one’s personal life, enhanced social involvement, and an appreciation for the collective purpose that accompanies advocacy work with VANDU. People who use drugs or those with lived and living experience are also often mourning the loss of close loved ones in their community from drug use; however, unlike bereaved mothers, their advocacy may be challenged even more because by coming forward and admitting their drug use, they are in fact admitting to something that, at present, may result in a criminal conviction (Knopf, 2012).

A key strength to this study is that, to our knowledge, our research addressing the advocacy of bereaved mothers engaged in drug policy reform is the first of its kind in Canada. We do, however, acknowledge several limitations to our research study. First, although we successfully included families from across various regions in Canada, both urban and rural, our sample was primarily women who would identify as “White” and “middle class.” Thus, a distinct limitation was our lack of representation of Indigenous families who we know are disproportionately affected by opioid-related deaths in Canada as a result of systemic racism, colonization, intergenerational trauma, and a lack of access to culturally safe care (First Nations Health Authority, 2017; Government of Alberta, 2017). In recruiting participants affiliated with our two partner organizations, our convenience sampling strategy did not result in bereaved mothers who identified as being from racialized groups and we spoke to very few women living in poverty or experiencing other forms of structural vulnerability. We see this limitation as being important to address in future studies, as to whether bereaved mothers from socially marginalized groups are more “silenced” than others and can experience significant barriers to advocating for drug policy change. Second, we also recognize that most mothers we spoke to had a child whose death was attributable to opioid use. Mothers bereaved from alcohol or stimulant use may advocate for different measures and be personally impacted by advocacy in a different way as a result. Finally, interviews took place during a time where there was a lot of public and political attention on addressing the overdose epidemic and saving lives. We recognize that the advocacy experience is specific to a defined time period and its cultural and political context regarding supporting people who use drugs.

Many of the bereaved mothers in our study spoke of being motivated by tremendous anger and disappointment in the health care system that was supposed to keep their child alive and healthy. Smith et al. (2018) alluded to this as the “professional silences” experienced by the mothers in their study who were trying to support their adolescent children being treated for substance use disorder. Therefore, our findings underscore the need for governments and service providers to support a family-centered approach to help individuals who have a loved one struggling with substance use. Despite evidence highlighting the importance of family inclusion in service delivery that challenges the current discourse based on an individualistic and biomedical model (McCune et al., 2017), such programs appear to be exceedingly rare (Ventura & Bagley, 2017). We also call for enhanced clinical support and resources for families grieving the passing of a loved one from substance-related causes. Essential resources, such as “Gone Too Soon” (British Columbia Centre on Substance Use [BCCSU], 2019), need to be distributed widely as they help people recognize the unique type of grief associated with substance-related deaths and aim to offer practical suggestions to those who require this support. For those who choose to use advocacy during their grieving process, health care providers, policymakers, researchers, and other community partners are encouraged to recognize and support the full and meaningful involvement of family members to help drive changes in our health care and justice systems.

Opportunities for people who use drugs and bereaved mothers to work together as allies in supporting one another in advocacy initiatives should be further explored. Also, advocacy group members themselves need to continue to support one another in ensuring that new and continuing members fully understand both the personal and collective benefits, as well as the challenges in becoming involved in such activities. We anticipate that this study’s findings will be of use to all organizations working to advance drug policy reform. It is hoped that, by examining and reflecting on the motivations and personal impact of bereaved mothers’ advocacy, all advocates will be provided with the additional working knowledge necessary to continue their efforts to bring about sustainable system reform.

## Supplemental Material

sj-docx-1-qhr-10.1177_10497323211006383 – Supplemental material for “It’s a Bit of a Double-Edged Sword”: Motivation and Personal Impact of Bereaved Mothers’ Advocacy for Drug Policy Reform Supplemental material, sj-docx-1-qhr-10.1177_10497323211006383 for “It’s a Bit of a Double-Edged Sword”: Motivation and Personal Impact of Bereaved Mothers’ Advocacy for Drug Policy Reform by Heather Morris, Elaine Hyshka, Petra Schulz, Emily Jenkins and Rebecca J. Haines-Saah in Qualitative Health Research

sj-docx-2-qhr-10.1177_10497323211006383 – Supplemental material for “It’s a Bit of a Double-Edged Sword”: Motivation and Personal Impact of Bereaved Mothers’ Advocacy for Drug Policy Reform Supplemental material, sj-docx-2-qhr-10.1177_10497323211006383 for “It’s a Bit of a Double-Edged Sword”: Motivation and Personal Impact of Bereaved Mothers’ Advocacy for Drug Policy Reform by Heather Morris, Elaine Hyshka, Petra Schulz, Emily Jenkins and Rebecca J. Haines-Saah in Qualitative Health Research

## Notes

1. In instances where only one participant is from a province, the name of that province is withheld to protect anonymity
