# Supplementary material for: “It’s a Bit of a Double-Edged Sword”: Motivation and Personal Impact of Bereaved Mothers’ Advocacy for Drug Policy Reform
Source: Qual Health Res. 2021 Sep 16;31(10):1812–22. doi: 10.1177/10497323211006383 (PMC8446895; doi:10.1177/10497323211006383)
Supplement: sj-docx-1-qhr-10.1177_10497323211006383 – Supplemental material for “It’s a Bit of a Double-Edged Sword”: Motivation and Personal Impact of Bereaved Mothers’ Advocacy for Drug Policy Reform [file sj-docx-1-qhr-10.1177_10497323211006383.docx]

Table 1: Participant Characteristics

| **Age**  Missing  65+  55-64  45-54  35-44 | 11  6  16  8  2 |
| --- | --- |
| **Household Income**  Less that $10,000  $10,000-$50,000  $50,000-$100,000  $100,000 +  Missing | 1  11  14  16  1 |
| **Marital Status**  Widowed  Single  More than 1 answer provided  Common-Law  Separated/Divorced  Married | 1  3  4  3  10  22 |
| **Regional Representation**  British Columbia  Alberta  Other Prairies  Ontario & Maritimes | 17  12  4  10 |
| **Child’s Gender**  Female  Male | 10  35 |
| **Year of Child’s Passing/Death**  2017  2016  2015  2014  2013  2012  Earlier than 2012  Unclear | 5  17  5  7  3  4  3  1 |
| **Child’s Age at Passing/Death**  40+  30-39  25-29  20-24  17-19 | 3  9  15  14  4 |
| **Was child’s passing/death a result of drug poisoning? (‘Overdose’ or ‘tainted drugs’ to some people)**  No  Yes  Unknown | 3  39  3 |
| **What substance(s) caused the passing/death?**  Carfentanil  Other (Suicide or N/A)  Methadone  Unknown  Other opioid (e.g. hydromorphone, morphine, heroin)  Polysubstance  Fentanyl | 1  2  2  3  3  14  20 |
