# Supplementary material for: “It’s a Bit of a Double-Edged Sword”: Motivation and Personal Impact of Bereaved Mothers’ Advocacy for Drug Policy Reform
Source: Qual Health Res. 2021 Sep 16;31(10):1812–22. doi: 10.1177/10497323211006383 (PMC8446895; doi:10.1177/10497323211006383)
Supplement: sj-docx-2-qhr-10.1177_10497323211006383 – Supplemental material for “It’s a Bit of a Double-Edged Sword”: Motivation and Personal Impact of Bereaved Mothers’ Advocacy for Drug Policy Reform [file sj-docx-2-qhr-10.1177_10497323211006383.docx]

**DRAFT INTERVIEW GUIDE (REVISED SEPT. 7 2017)**

- To be refined following pilot interviews and team consultation
- Underline + CAPS = General topics to probe

**Pre-interview process**:

- Review study aims
- Review and complete Informed Consent (walk through form)
- Participants ask questions or seek clarifications prior to signing
- Complete Brief Survey
- Complete Receipt form for remuneration ($50)

**Grand tour question**: Before we get started with the focus of our interview today, why don’t you tell me a bit about what is going on in your life right now?

**Opening question**: As you know, the focus of our interviews is to learn from the experiences of mothers in sharing their stories – their personal and family story – of substance passing as a tool for advocacy. Imagining that we’ve just met, and I don’t know anything about you, can you tell me your story? ***Where does the story begin for yo***u?

**ADVOCACY** *(General probe: Let’s talk about why you share your story; how you became an advocate?)*

- How/when did you first decide to share your story/your family's story around substance passing?
- How did you first get connected to or involved with the group (mumsDU/Moms Stop the Harm)?
- What does advocacy or ‘being an advocate’ mean to you?
- Was this the first time in your life you've done something like this (gone public, became involved in advocacy/activism)?
- In addition to sharing your story - what other different types of activities or advocacy work are you involved in? (concrete examples)
- Why do you think some bereaved parents engage in advocacy, when others do not?

**MESSAGES** *(General probe: What are the messages you hope to get across through your work?)*

- By sharing your story and doing this advocacy work, what do you want people to know about your child? About your personal and family experience?
- Why is it important for mothers in particular, to deliver these messages, to speak out on this issue?
- What are the central messages or "themes" you hope to get out there through sharing your story?
- What types of changes do you hope this will bring about?
- Do you think/have you seen that mothers and families sharing their stories has brought about changes?
- What are the impacts/effects that you have seen from this work (on other individuals, organizations, government)?

**MEDIA** *(General probe: Tell me about your experiences engaging with media?)*

- How do you feel about how the media represented your story? Your child?
- What are your thoughts on the ways that media represents stories of substance passing and people who use substances (i.e. what do you like, not like; what do you react to?)
- What about the ways that other people/groups (i.e. politicians) have taken up and shared your story?
- What are the negative effects of doing media interviews (i.e. reporters wanting interviewees to ‘relive’ the story of loss to make for a ‘better’ story).

**PERSONAL - IMPACT/COSTS** *(General probe: How has sharing your story affected you?)*

- What have been the effects on you, personally, from sharing this story? (positive and negative)
- On your immediate family? Social networks?
- What are the drawbacks/personal and social costs to doing this type of advocacy work?
- Do you ever get tired of sharing your story?
- Do people tell you that you shouldn’t share it?
- Are there parts of the story you don't share, or that you choose to keep private?
- If you have other children, can you talk about how you’ve addressed the issue of substance use with them, since your child died? Did anything change in that conversation or approach?
- Have you received feedback from people and ‘the public’? What type? How has the response from the general public may impacted you personally? How has it shaped ongoing advocacy work?

**GRIEVING AND ADVOCACY** (*What is the impact of advocacy on the grief process?)*

- What are the effects of advocacy on the grief process? Does it help?
- Does advocacy prolong the grief process? (i.e. compassion fatigue, “keeping the wounds open,” preventing people from moving on)
- How is advocacy a way of keeping connected to a child that had died? Does doing advocacy work “replace” the work of trying to save one’s child?
- What are the effects of doing advocacy work on a marriage or relationship; siblings and the family?

*What services have you accessed or tried to access for support (e.g. supports and/or counselling) for yourself or other family members before or after the death of your child"

**Closing**: Is there anything else you would like to add that we haven’t discussed?
